# The effect of symptomatic extra-articular manifestations of rheumatoid arthritis on disease activity and patient-reported outcomes: a cross-sectional and observational study

**DOI:** 10.1186/s42358-026-00569-5

**Published:** 2026-07-25

**Authors:** Ahmet Oruç, Hasan Satış, Merve Oruç, Reyhan Bilici, Abdurrahman Tufan, Mehmet Akif Öztürk

**Affiliations:** 1 Department of Medical Oncology, Necmettin Erbakan University, Konya, Turkey; 2 Department of Rheumatology, Dr. Abdurrahman Yurtaslan Ankara Oncology Training and Research Hospital, Ankara, Turkey; 3 Department of Nephrology, Necmettin Erbakan University, Konya, Turkey; 4 Division of Rheumatology, Department of Internal Medicine, Necmettin Erbakan University, Konya, Turkey; 5 Division of Rheumatology, Department of Internal Medicine, Gazi University Faculty of Medicine, Ankara, Turkey

**Keywords:** Rheumatoid arthritis, Extra-articular involvement, Disease activity

## Abstract

**Introduction:**

Rheumatoid arthritis (RA) is a chronic inflammatory disease that primarily affects the joints but also has extra-articular manifestations. Extra-articular manifestations (EAMs) in RA encompass a wide range of clinical findings that may significantly affect patients’ quality of life. This study aimed to compare daily life, social function, and work productivity between RA patients with and without EAMs. In this study, we aimed to determine the differences in daily life, social life, and work between patients with and without EAM.

**Methods:**

This cross-sectional and observational study included 402 RA patients (2010 ACR-EULAR). EAM was defined as systemic symptoms and organ involvement occurring outside of the joint findings caused by RA. The presence of EAM was grouped as symptomatic/asymptomatic. Patients were evaluated once during the study period in an outpatient setting. Patients completed the Health Assessment Questionnaire (HAQ), Short Form-36 (SF-36), and Work Productivity and Activity Impairment: General Health (WPAI: GH) forms under clinician observation. Continuous variables were compared using the Mann–Whitney U test and Independent Samples T-test, while Fisher’s exact test or chi-square test was used for categorical variables. The significance level was set at 5% (*p* < 0.05).

**Results:**

The study included 402 patients. 325 (80.8%) were female, with a median (IQR) age of 56 (47–64.25) and a mean±standard deviation (SD) disease duration of 9.7 ± 8.6 years. EAM was present in 200 patients. Patients were classified as having symptomatic EAM (sEAM) (n = 81), asymptomatic EAM (asEAM) (n = 119), and no EAM (n = 202). The median age was 60 (54.5–67), and the median age at diagnosis was 49 (39.5–58) in the sEAM group, which had the highest values (p:<0.001 and p:0.003, respectively). Peripheral neuropathy detected in 24 (29.6%) patients was the most common symptomatic EAM, while keratoconjunctivitis sicca detected in 44 (36.9%) patients was the most common asymptomatic EAM. In sEAM patients, HAQ mean ± SD was 0.92 ± 0.73, while in non-EAM patients it was 0.68 ± 0.65, p = 0.021. In SF-36 results, physical function, social function, role physical, vitality, and mental health were statistically significantly lower in patients with sEAM compared to other groups. The percentage of working patients was higher at 28.2% in those without EAM, p:0.010. Absenteeism, mean ± SD, was higher in the sEAM group at 12.8 ± 28.1, p: 0.037, and activity impairment was also higher in sEAMs at mean ± SD 44.2 ± 29.1, p:0.025.

**Conclusions:**

Regardless of RA disease activity, patients with sEAM experience negative impacts on both their daily and social lives as well as their work life. Symptomatic EAMs are associated with significant impairment in quality of life and work productivity, independent of traditional disease activity measures. Clinicians should therefore systematically assess and manage these manifestations as an integral component of RA care.

## Introduction

Rheumatoid arthritis (RA) is a chronic, systemic, inflammatory disease of unknown etiology. Although RA primarily targets synovial tissues, it can also affect multiple systems. Its prevalence varies among populations but is generally between 0.5% and 1% [1]. Although RA primarily affects the joints, it is increasingly recognized as a systemic disease given its association with a wide spectrum of extra-articular manifestations (EAMs) such as rheumatoid nodules, neuropathy, pericarditis, pulmonary involvement, and vasculitis [2]. Persistent systemic inflammation is a well-known risk factor for the development of EAM and is frequently associated with increased disease severity and mortality [3]. There is no common definition or usage in studies on extra-articular manifestations in rheumatoid arthritis, and the differences in the findings presented in the studies limit comparability [4]. EAM was detected in an average of 35–40% of the studies, and in addition, at least one lesion was detected in 46% of patients during 30 years of follow-up [5].

It is well known that RA not only causes physical problems, but also affects patients emotionally and psychologically. Stiffness and pain in the joints have been shown to have a significant impact on patients’ social and family lives [6]. Beyond its impact on family and social life, RA frequently impairs patients’ ability to perform professional duties, contributing to reduced work productivity, absenteeism, and early retirement [7]. Several validated instruments are available to assess quality of life and work productivity in patients with RA, including the Health Assessment Questionnaire (HAQ), Short Form-36 (SF-36), and Work Productivity and Activity Impairment questionnaire (WPAI: GH) [8]. While these scales primarily focus on RA disease activity, information on the effect of EAMs on these parameters is limited.

In our study, findings not directly related to the locomotor system and excluding complications and symptoms related to RA treatment or other comorbidities were evaluated as EAM. Since the quantitative effect of EAMs on composite outcomes reported by patients, particularly on work productivity (e.g., WPAI-GH), has not been sufficiently clarified, we aimed to investigate all EAMs in a large cohort during the course of RA, examine the effect of EAMs on disease activity, and assess their impact on patients’ daily lives and work.

## Methods

### Participants

This cross-sectional and observational study was conducted between June 2020 and June 2021. Patients aged 18 years and older who had previously been diagnosed with rheumatoid arthritis according to the 2010 ACR/EULAR diagnostic criteria were evaluated [9]. Patients with an uncertain RA diagnosis or secondary rheumatic disease, patients who did not consent to participate, and patients who were unable to complete the required form were excluded from the study. Patients were evaluated once during these 12 months. Every eligible patient was included in sequence.

### Measurements

Patients included in the study were evaluated in outpatient settings. Physical examinations of patients were performed by the investigators. Clinical and demographic data were obtained from the hospital database. Afterwards, patients were asked to answer the PaGA (Patient Global Assessment, 0–10 scale) [10], Fatigue (0–10 scale) [11], Functional status was assessed using the Health Assessment Questionnaire (HAQ), with scores ranging from 0 (no disability) to 3 (severe disability) [12, 13], Visual Analogue Scale (VAS,0–10 scale) [14], Health-related quality of life was evaluated using the Short Form-36 (SF-36) [15], and Work productivity and activity impairment were measured using the Work Productivity and Activity Impairment questionnaire for general health (WPAI: GH) [16, 17]. Disease activity was calculated using the DAS-28-ESR and DAS-28-CRP [18, 19].

### Extra-articular manifestations (EAMs)

EAMs were identified through systematic review of medical records, physical examination, and available diagnostic tests (e.g., chest imaging, pulmonary function tests, ophthalmologic evaluation, nerve conduction studies). Each EAM was diagnosed according to established clinical criteria. The classification of symptomatic or asymptomatic EAM is based on symptoms reported by the patient and recorded during the evaluation. Osteoporosis, osteopenia, myelopathy, thrombocytosis, lymphopenia, leukemia, cardiac involvement other than pericardial effusion, drug-induced nephrotoxicity, and secondary amyloidosis, which are considered complications, are not included as EAM.

Considering that not all EAMs cause symptoms in patients, EAMs were categorized as symptomatic and asymptomatic. The EAMs identified in our cohort in Table 1 are classified both according to the involvement system and as symptomatic (s)/asymptomatic (as).

### Statistics

All statistical evaluations were performed in the IBM Statistical Package for Social Sciences (SPSS) 25.0 package program. The normal distributions of the variables were analyzed by histogram plots, mean and standard deviation ratios, and analytical methods (Kolmogorov-Smirnov and Shapiro-Wilks). Categorical variables were expressed as numbers and percentages (%), and descriptive data were expressed as mean±standard deviation if normally distributed, and as median, minimum, maximum values, and interquartile range (IQR) if not normally distributed. Chi-square test was used to analyze categorical variables. An independent samples t-test was used between two independent variables with normal distribution, and mann whitney u test was used between two independent groups without normal distribution. In all tests, *p* < 0.05 was considered statistically significant.

## Results

A total of 402 patients were included in the study. Of these, 325 (80.8%) were female, and 77 (19.2%) were male, with a median age (IQR) of 56 (47–64.25). The median age (IQR) at the time of rheumatoid arthritis diagnosis was 45.4 (35–55), and the mean disease duration was 9.7 ± 8.6 years.

**Table 1:** Classification of EAM according to systems and symptom status

	Symptomatic EAMs	Asymptomatic EAMs
**Skin involvements**		
Rheumatoid nodule, n	10	33
Purpura, n	-	3
Raynaud’s phenomenon, n	4	13
Livedo reticularis, n	-	1
Skin ulcers, n	7	-
Leg ulcers, n	6	-
Neutrophilic dermatitis, n	1	-
**Neurological involvements**		
Entrapment neuropathy, n	14	-
Peripheral neuropathy, n	24	-
**Eye involvements**		
Keratoconjunctivitis sicca, n	5	44
Episcleritis, n	2	-
Scleritis, n	2	-
Peripheral ulcerative keratitis, n	3	-
**Pulmoner involvements**		
Pulmoner rheumatoid nodule, n	2	20
Pleural effusion, n	12	3
Usual interstitial pneumonia, n	9	-
Non-specific interstitial pneumonia, n	15	-
Organized pneumonia, n	2	-
Pulmonary hypertension, n	-	2
**Hematologic involvements**		
Chronic disease anemia, n	-	60
Leukopenia, n	-	11
Lymphadenopathy, n	-	7
Lymphoma, n	-	1
**Renal involvements**		
Nephrotic syndrome, n	-	4
Glomerulonephritis, n	-	3
**Cardiac involvement**		
Pericardial effusion, n	1	2

EAM was present in 200 patients. Of these patients, 81 had symptomatic EAM while 119 had asymptomatic EAM.

**Table 2:** Demographic characteristics and disease-related conditions in RA patients according to EAM status

	*P* with sEAM (*n*:81)	*P* with asEAM (*n*:119)	*P* without EAM (*n*:202)	*p* value
Gender (male/female), *n* (%)	22 (27.2) /59 (72.8)	14 (11.8) /105(88.2)	41(20.3) /161(79.7)	0.021
Age, median (IQR)	60 (54.5–67)	59 (49–66)	52.5 (40.75-62)	< 0.001
Age at diagnosis, median (IQR)	49 (39.5–58)	46 (35.5–58.1)	43 (32–53)	0.003
Duration of RA years, mean ± SD	10.9 ± 10.8	10.7 ± 9.3	8.7 ± 7	0.324
Number of SP patients, n (%)	63 (77.8)	93 (78.2)	151 (74.8)	0.744
Never smoking, n (%)	31 (38.3)	56 (47.1)	95 (47)	0.354
With comorbidity, n (%)	62 (76.5)	82 (68.9)	111(55)	< 0.001
BMI, median (IQR)	28.5 (25.7–32.7)	27.4 (23.4–30.8)	26.5 (22.9–29.7)	0.001

P: Patient, sEAM: Symptomatic Extra-Articular Manifestation, asEAM: Asymptomatic Extra-Articular Manifestation,

RA: Rheumatoid Arthritis, BMI: Body mass index, SD: Standard deviation, IQR: Interquartile range, SP: Seropositive

The proportion of male patients was higher in those with symptomatic EAM (27.2% vs. 11.8%), *p* = 0.021. The median age of the symptomatic EAM group was 60 (54.5–67), while the median age of the non-EAM group was 52.5 (40.75-62), *p* < 0.001. The median age at diagnosis was 49 (39.5–58) in the symptomatic EAM group and 43 (32–53) in the non-EAM group, *p* = 0.003. Although there was a numerical difference in RA duration between the groups, there was no statistical difference. Comorbidity and high BMI were higher in the asymptomatic EAM group. Clinical characteristics according to the presence of EAM and symptomatic status are shown in Table 2.

As shown in Table 1, cutaneous involvement was found in a total of 67 (16%) patients. Rheumatoid nodules were seen in 43 patients (10.7%) and Raynaud’s phenomenon in 17 patients (4.2%). In one (0.2%) patient with neutrophilic dermatitis, there was no other cutaneous involvement, and the diagnosis was made by biopsy. Livedo reticularis was found in one (0.2%) patient. Skin and leg ulcers were seen simultaneously in four patients. Purpura was present in three (0.7%) patients, and two of these patients had no additional cutaneous involvement detected.

Lung involvement in rheumatoid arthritis was detected in 53 (13%) patients. The findings were evaluated by computed tomography, pulmonary function test and transthoracic echocardiography. Rheumatoid nodules in the lung were seen in 22(5.5%) patients. Of the interstitial involvement patterns, UIP was seen in 9 (2.2%) patients and NSIP in 15 (3.7%) patients. Two of the 15 (3.7%) patients with pleural effusion also had rheumatoid nodules in the lung. NSIP and rheumatoid nodules were seen together in 3 patients, and UIP and rheumatoid nodules were seen together in 2 patients. Two patients with pulmonary hypertension had concurrent UIP.

Extraarticular eye involvement was detected in 54 (13.9%) RA patients. 49 (12.2%) patients had keratoconjunctivitis sicca, the most common form of involvement. Despite the use of tear solution, 5 patients remained symptomatic and were included in the sEAM group. 41 (10.1%) patients with neurologic extra-articular involvement were identified. In 14 (3.5%) of these patients, electromyography was performed, and entrapment neuropathy was detected.

Pericardial effusion was detected in 3 (0.7%) patients, and no additional cardiac involvement was observed in these patients. The clinical picture did not develop in any patient. Extraarticular hematological involvement was detected in a total of 79 (19.6%) patients. The most common of these involvements is anemia of chronic disease, which was found in 60 (14.9%) patients. In the study, the presence of anemia was detected in a total of 110 patients. However, since iron deficiency, thalassemia, vitamin B12, and folic acid deficiency played a role in the etiology of the remaining 50 patients, EA was not considered as involvement. Only one of the 7 (1.7%) patients with lymphadenopathy had anemia of additional chronic disease. Lymphoma was seen in one patient and is in remission.

Renal pathologies as extra-articular involvement were detected in a total of 7 (1.7%) patients. The most common condition was nephrotic syndrome, which was detected in 4 (1%) patients. Glomerulonephritis 3 (0.7%) was observed.

**Table 3:** Patient assessment of RA activity and clinic parameters

	*P* with sEAM (*n*:81)	*P* with asEAM (*n*:119)	*P* without EAM (*n*:202)	*p* value
HAQ mean ± SD	0.92 ± 0.73	0.75 ± 0.8	0.68 ± 0.65	0.021
VAS mean ± SD	4.61 ± 3	5.05 ± 3.28	4.33 ± 2.98	0.151
Fatigue mean ± SD	4.91 ± 3.1	5.11 ± 2.93	4.4 ± 2.8	0.089
PaGA mean ± SD	3.37 ± 2.8	4.24 ± 3	3.74 ± 2.95	0.049
ESR, median (IQR)	27 (15.25–39.75)	28.5 (15–38)	20 (13–29)	< 0.001
CRP, median (IQR)	5 (3-13.5)	6 (3–12)	4 (1–2)	0.023
DAS 28 ESR mean ± SD	2.95 ± 1	3.18 ± 1.05	2.8 ± 1.09	0.003
DAS 28 CRP mean ± SD	2.44 ± 0.9	2.65 ± 1.03	2.39 ± 1.01	0.037

VAS: Visual analog scale, PaGA: patient global assessment, SD: Standard deviation

In sEAM patients, HAQ mean ± SD was 0.92 ± 0.73, while in non-EAM patients it was 0.68 ± 0.65, *p* = 0.021. PaGA mean ± SD was 3.37 ± 2.8 in symptomatic EAM patients, while it was 4.24 ± 3 in asymptomatic EAM patients, *p* = 0.049. ESR, CRP, DAS-28 ESR, and DAS-28 CRP were higher in asymptomatic EAM patients compared to symptomatic EA patients and non-EAM patients. This is shown in detail in Table 3.

**Table 4:** SF-36 results according to EA status

	*P* with sEAM (*n*:81)	*P* with asEAM (*n*:119)	*P* without EAM (*n*:202)	*p* value
Physical Function (PF), median (IQR)	65 (45–85)	70 (47.5–90)	75 (55–95)	0.050
Social function (SF), median (IQR)	62.5 (50-87.5)	75 (50–100)	75 (50–100)	0.011
Role Physical (RP), mean ± SD	38.6 ± 44.9	43.2 ± 45.7	52.8 ± 45.6	0.007
Role Emotional (RE), mean ± SD	54.7 ± 46.3	55.4 ± 46.9	60.8 ± 45	0.305
Mental Health (MH), mean ± SD	61.5 ± 18.4	64 ± 18.3	65.8 ± 18.9	0.048
Vitality, mean ± SD	46.7 ± 21.8	48.5 ± 22.9	54.4 ± 23.1	0.004
Body Pain (BP), mean ± SD	55.8 ± 30.2	49.8 ± 28.7	60 ± 30.1	0.012
Global Health (GH), median (IQR)	45 (30–65)	50 (30–70)	50 (35–70)	0.292

SD: Standard deviation

In SF-36 results, PF, SF, RP, vitality, and mental health were statistically significantly lower in patients with sEA compared to other groups. BP was higher in patients without EAM (60 ± 30.1), while GH was lower in those with sEA. Table 4 shows the SF-36 results in detail.

**Table 5:** WPAI: GH results according to EA status

	*P* with sEAM (*n*:81)	*P* with asEAM (*n*:119)	*P* without EAM (*n*:202)	*p* value
Number of working patients, *n* (%)	15 (18.5)	17 (14.3)	57 (28.2)	0.010
Absenteeism, mean ± SD	12.8 ± 28.1	11.2 ± 14.1	6.4 ± 20.7	0.037
Presenteeism, mean ± SD	38.8 ± 29.1	33.5 ± 31	28 ± 30.9	0.268
Work productivity loss, mean ± SD	43.8 ± 29.5	37.2 ± 35.7	28.8 ± 31.6	0.148
Activity impairment, mean ± SD	44.2 ± 29.1	43.2 ± 32	35.3 ± 29	0.025

SD: Standard deviation

As shown in Table 5, the percentage of RA patients without EAM was higher at 28.2% compared to those with EAM, p:0.010. When considering working patients, absenteeism was highest among symptomatic EAM patients, with a mean ± SD of 12.8 ± 28.1, p:0.037. Presenteeism and loss of work productivity were numerically higher in symptomatic EAM patients. However, the group with the highest activity impairment was symptomatic EAM patients, 44.2 ± 29.1, *p* = 0.025.

## Discussions

In our study, we determined the frequency of extra-articular manifestations in rheumatoid arthritis, which affects all systems, in a relatively large cohort, and whether EAMs cause symptoms. We found that symptomatic EAMs negatively affect patients’ daily and social lives, as well as their work, regardless of disease activity.

We evaluated RA EAMs separately for all systems; nevertheless, to better understand whether EAMs affect patients’ quality of life, we divided them into two groups based on whether they were symptomatic or not. As in previous studies [20], rheumatoid nodules were the most common finding in cutaneous EAMs, and one in four patients was symptomatic. While the presence of purpura and livedo reticularis did not cause symptoms in the majority of patients with Raynaud’s phenomenon, vasculitis and neutrophilic dermatitis did cause symptoms. Compression neuropathy and peripheral sensory neuropathy were the two most commonly observed nervous system involvement patterns that caused symptoms [21]. In our study, keratoconjunctivitis sicca was the most common ocular EAM [22]. However, the vast majority of these patients remained asymptomatic with the use of tear solutions. The presence of episcleritis, scleritis, and peripheral ulcerative keratitis significantly affected patients’ daily lives. In the lungs, the most common form of involvement was the rheumatoid lung nodule. Unlike previous studies, the prevalence of NSIP was higher than that of UIP in our study [23]. It should not be overlooked that patients with pericardial effusion will experience symptoms. Hematologic EAMs did not cause symptoms in patients. It should be noted that this condition is associated with a chronic process, and patients may not describe it as a new symptom [24].

In patients with asEAM, the elevated levels of ESR, CRP, DAS-28 ESR, and DAS-28 CRP may suggest higher RA activity in this group of patients, both laboratory and clinically. Patients with asymptomatic EAM reported higher patient global assessment (PaGA) scores compared to those with symptomatic EAM (*p* = 0.049), despite having higher disease activity indices. This finding suggests that patient perception of disease burden may be influenced by factors beyond traditional disease activity measures. The fact that the HAQ score is statistically higher in the same group of patients compared to the other two groups indicates that sEAMs affect daily life.

When evaluating the SF-36 results, the lower scores in physical and social functioning among those with sEAM may explain the lower vitality, mental health, and global health in this group of patients. From the patients’ perspective, the presence of EAM, which causes persistent symptoms, appears to have affected quality of life more than pain.

The relationship between RA disease activity and work life has been evaluated. However, our study is important in terms of examining the effect of EAM presence and the presence or absence of symptoms on work life [25]. The number of patients without EAM was 57 (28.2%), which was statistically significantly higher than that of patients with EAM. When evaluated using SF-36 results, physical and functional limitations may have affected patients’ working lives. It is important to note that absenteeism and its activity impairment are statistically higher in patients with sEAM, and that presenteeism and loss of work productivity are also increased in these patients. Considering that disease activity indicators such as DAS-28 are high in those with asEAM and body pain is high in the group without EAM, the presence of symptomatic EAM appears to have a greater impact on working life.

Our study had some limitations. Due to the cross-sectional design, it is not possible to draw conclusions about causality between EAMs and the outcomes. Although EAMs are classified as symptomatic or asymptomatic, this distinction may inherently involve subjectivity, and this should be kept in mind. Excluding patients who were unable to complete the forms may have led to the systematic exclusion of patients with greater functional impairment or those experiencing cognitive difficulties. Despite all this, we tried to include every sequentially eligible patient. We tried to reduce bias in this way.

In this cross-sectional study, symptomatic extra-articular manifestations were associated with significant impairments in functional status (HAQ), quality of life (SF-36), and work productivity (absenteeism, activity impairment), independent of traditional disease activity measures. In contrast, asymptomatic EAMs were associated with higher disease activity scores but not with worse patient-reported outcomes. These findings underscore the importance of systematically assessing the symptom status of EAMs in clinical practice, as their impact on patients’ lives extends beyond the traditional paradigm of disease activity.

## Data Availability

No datasets were generated or analysed during the current study.

## Abbreviations

RA: Rheumatoid arthritis
EAMs: Extra-articular manifestations
HAQ: Health Assessment Questionnaire
SF-36: Shorts form-36
VAS: Visual analogue scale
PaGA: Patient global assessment
WPAI: Work productivity and activity impairment
GH: General health
